# Supplementary material for: Transcriptome Analysis Reveals Modulation of Human Stem Cells from the Apical Papilla by Species Associated with Dental Root Canal Infection
Source: Int J Mol Sci. 2022 Nov 20;23(22):14420. doi: 10.3390/ijms232214420 (PMC9695896; doi:10.3390/ijms232214420)
Supplement: Supplementary file 1 [file ijms-23-14420-s001.zip › ijms-2002416-supplementary.pdf]

Supplementary Material

A

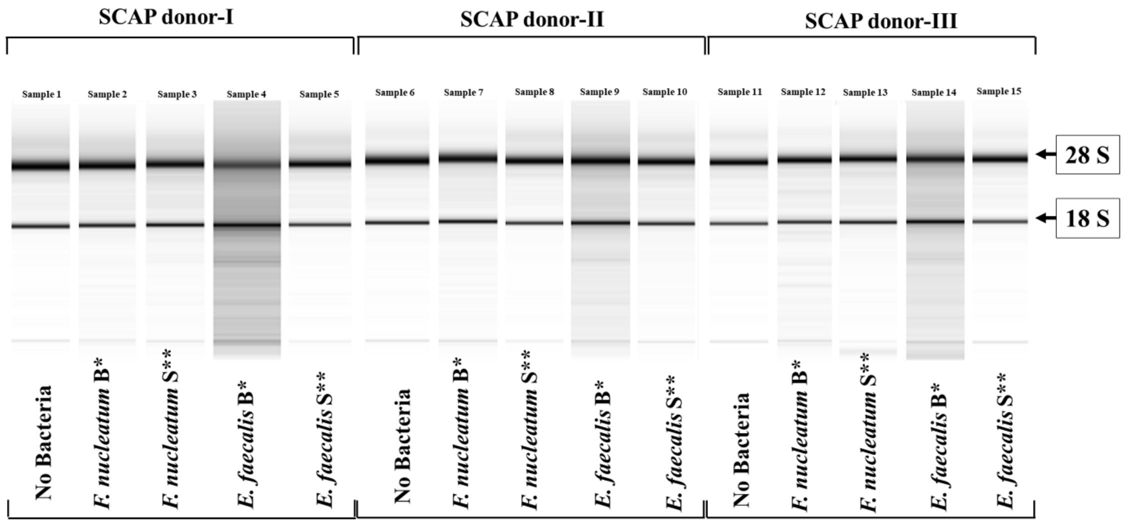

B

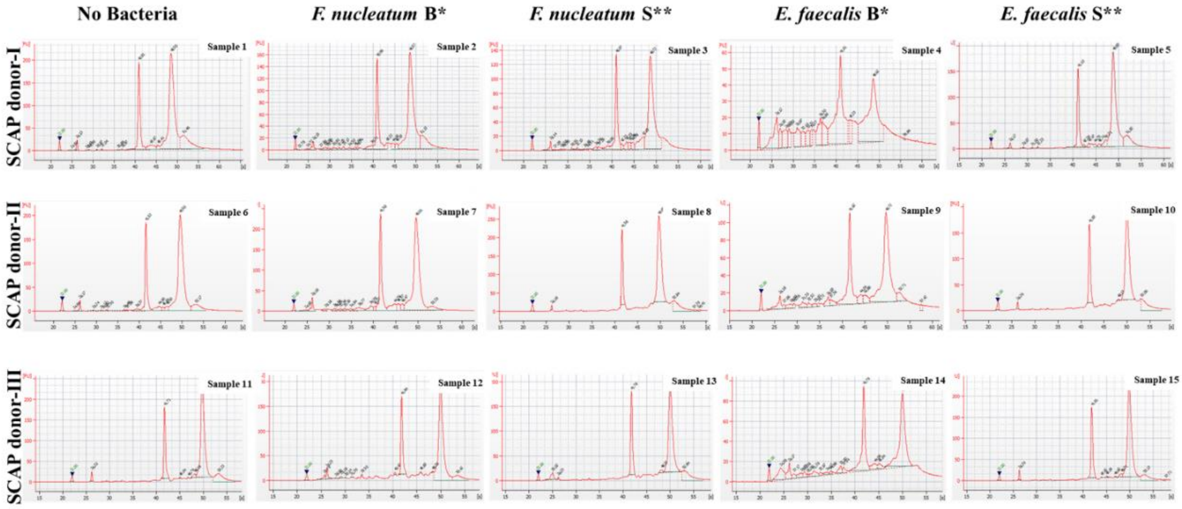

Figure S1. Data summary of all extracted RNA samples obtained with the tests on the Agilent 2100 instrument: data presented in a gel-like view (**A**); data presented as the electropherogram view (**B**). *B: bacteria; **S: supernatant.

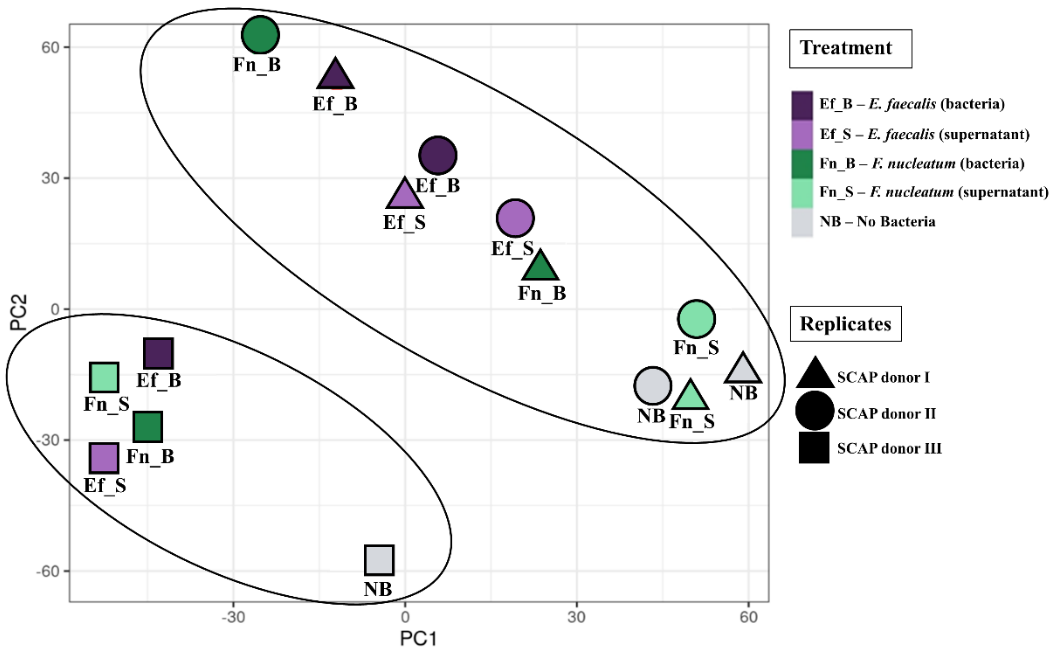

Figure S2. Principal component analysis (PCA) results prior to the batch correction.

Table S1. Identification of clinical isolates.

Clinical isolate	Isolate identity with strain	Initial identification		Confirmation of initial identification	Re-identification	
		16S rRNA, % match		**MALDI-TOF score**	16S rRNA, % match	
		FW	R		FW	R
R9(50)	*Fusobacterium nucleatum subsp. polymorphum*	99,8	99,9	**1,95**	-	-
4 F	*Enterococcus faecalis*	99,7	100	**2,25**	-	-
